# Prediction of roof supporting pressure for shallow tunnels in layered soils incorporating the effect of pore water pressure

**DOI:** 10.1371/journal.pone.0217351

**Published:** 2019-06-13

**Authors:** Hong-tao Wang, Xiao-jing Li, Ping Liu, Xin Zhang, Lu-yao Liu

**Affiliations:** 1 School of Civil Engineering, Shandong Jianzhu University, Jinan, China; 2 Shandong Co-Innovation Center for Disaster Prevention and Mitigation of Civil Structures, Shandong Jianzhu University, Jinan, China; 3 State Key Laboratory of Water Resources and Hydropower Engineering Science, Wuhan University, Wuhan, China; Central South University, CHINA

## Abstract

In this study, we propose a method for predicting the supporting pressure required for shallow tunnels in layered soils, based on a curved roof collapse mechanism with multi-failure surfaces. In this method, the effect of the number and thickness of soil layers, pore water pressure, arbitrary roof profile, and ground additional load is considered simultaneously. A nonlinear power-law failure criterion is employed to describe the failure characteristics of the roof soils. The internal energy dissipation rate and the work rates produced by external forces are obtained based on the associated flow rule and plasticity potential theory. The analytical expressions of the required supporting pressure and roof collapse surfaces are obtained with the upper bound method. Furtherly, a shallow rectangular tunnel in two soil layers is selected for parametric investigation. The change laws of the required supporting pressure and collapse curves under varying parameters are obtained. Furthermore, the corresponding engineering recommendations are given, which may potentially provide references for the support design and construction of shallow tunnels in layered strata.

## Introduction

The tunnel, as an underground building, can not only effectively alleviate problems like road traffic congestion and land resource shortage, but also can bring enormous convenience to people's travel and transportation. At present, with the rapid development of the world economy and technology, various types of tunnels are increasingly scaled up in engineering fields including transportation, water conservancy and hydropower, urban subway, and underground mining. The tunnels may be built in the rocks or soils based on the stratum geological conditions. For the tunnels in the soils, due to generally low strength of the soil masses, intense deformation and failure may occur in the surrounding rock after tunnel excavation. In particular, if the tunnels are buried in shallow soils or affected by adverse conditions such as groundwater seepage and ground additional loading, it is very easy to cause land subsidence and even a landslide, seriously threatening the safety of the lives and property of people.

The safety and stability of the shallow tunnels in soils is a research hotspot of many scholars. Jin et al. [1] focused on the quality of ventilation in road tunnels with roof openings, and investigated the influences of vehicle speeds and roofopening layouts through 12 groups of experiments. Fan et al. [2] conducted a series of numerical simulations, and investigated the effect of strong environmental wind on a tunnel fire. Chambon and Corte [3], Kamata and Mashimo [4], Shin et al. [5], and Kirsch [6] conducted the laboratory tests to investigate the failure characteristics of the tunnel face in clay or sand. Leca and Dormieux [7], Augarde et al. [8], Lee et al. [9], Osman et a l. [10], Khezri et al. [11], and Zhang et al. [12] investigated the two or three dimensional stability of the tunnel face based on upper or lower bound theorems. Compared with other research approaches, the upper bound method can simplify the complex and tedious calculation, by constructing a kinematically admissible velocity field for the shallow tunnel failure without considering the stress equilibrium conditions. Meanwhile, the close-to-actual failure mechanisms for the tunnels can be deduced by using this method. Fraldi and Guarracino [13–14] initially introduced this method to analyze the roof collapse mechanisms for deep tunnels, and made comparisons with the results of numerical simulation to validate the effectiveness of the proposed method. Huang and Yang [15], Yang and Huang [16–17], Zhang et al. [18], Guan et al. [19], Qin et al. [20–21] further developed this theory. Particularly, Huang and Yang [15] introduced the effect of pore water into the collapse mechanism of a circular tunnel. Yang and Huang [16–17] presented the collapse mechanism of the shallow circular tunnel, and conducted three-dimensional upper bound limit analysis of the failure mechanism of a rectangular cavity. Zhang et al. [18] compared the proposed collapse mechanism with the results of model test. Guan et al. [19] proposed the failure mechanism of the supported cavity roof with arbitrary profile. Qin et al. [20–21] incorporated the influence of varying water table into the collapse mechanism, and proposed the roof collapse mechanisms for tunnels in layered strata. However, these researches were based on the non-linear Hoek-Brown failure criterion, and may be more suitable for tunnels in rock media. As for the tunnels in soil media, a failure criterion for soil mass should be chosen for analyzing. Besides, the natural soils typically have layered structures due to the influence of sedimentary process in strata, and this should not be ignored. Actually, Qin et al. [21], Yang and Yao [22] had introduced such influence into their researches, but these research findings mainly focused on the roof failure characteristics, and the required supporting pressure for maintaining the tunnel stability was less discussed.

According to the previous researches, we propose a prediction method of the supporting pressure required for shallow tunnels in layered soils in this paper, based on a curved roof collapse mechanism. The effect of the number and thickness of the soil layers, pore water pressure, roof profile, additional load on ground surface, and nonlinear failure characteristics of the soil masses is incorporated into the collapse mechanism simultaneously. The analytical solutions of the required supporting pressure and the roof collapse curves are derived using a nonlinear power-law failure criterion and the upper bound method. The research findings may serve as a guiding theory for the supporting design and construction of the shallow tunnels in layered soils.

## Nonlinear power-law failure criterion and its associated flow rule

**Fig 1:**
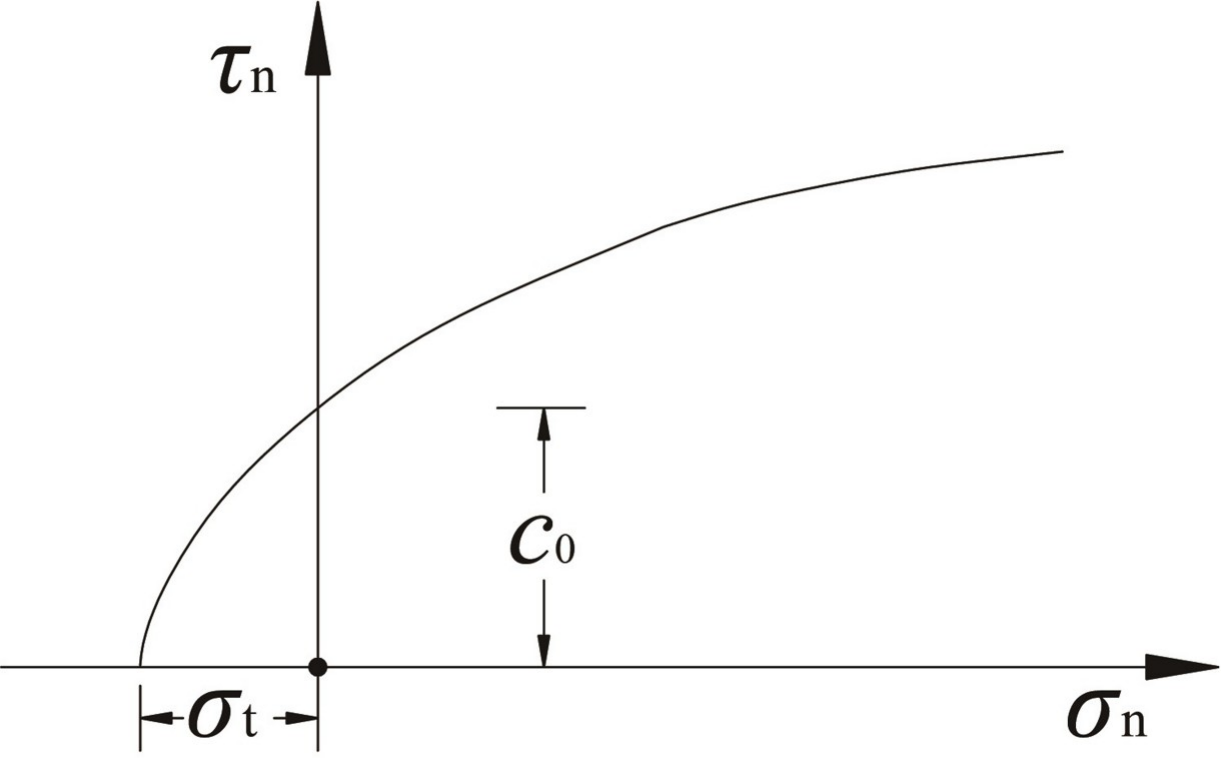
Nonlinear failure criterion in the Mohr plane $\sigma_{n}-\tau_{n}$.

Mohr-Coulomb failure criterion has been widely applied in geotechnical engineering due to its simplicity and effectiveness. In the expression of this criterion, the normal stress (*σ*_n_) and shear stress (*τ*_n_) at the failure surface are in linear relation in the Mohr plane *σ*_n-_*τ*_n_. However, previous studies [23–28] revealed that the soils present obvious nonlinear failure characteristics and the corresponding strength envelopes are approximately a convex curve. Based on this fact, the nonlinear failure criterion may be more suitable in analyzing the stability problems involving the deformation and failure of soils in engineering. Hence, we utilize a nonlinear power-law failure criterion [22, 25–26] to describe the failure characteristics of roof surrounding soil masses in this paper. The corresponding expression can be written as:

$$\tau_{\text{n}}=c_{0}{\left(1+\sigma_{\text{n}}/\sigma_{\text{t}}\right)}^{1/m}$$ (1)

where $c_{0}$ and $\sigma_{\text{t}}$ are the initial cohesion and tensile strength of the soil mass respectively, *m* is the dimensionless nonlinear coefficient (related to the properties of the soil masses) and m≥1. Fig 1 shows the strength envelope curve. Particularly, $\sigma_{\text{t}}$ and $c_{0}$ are the intercepts of the curve with the x-axis and y-axis, the magnitude of *m* indicates the curvature of the envelope.

The yield function *F* corresponding to the nonlinear power-law failure criterion in the Mohr plane $\sigma_{n}-\tau_{n}$ can be expressed as:

$$F=\tau_{\text{n}}-c_{0}{\left(1+\sigma_{\text{n}}/\sigma_{\text{t}}\right)}^{1/m}$$ (2)

Furtherly, we assume that the yield function *F* of the soil mass at failure is equal to the plastic potential function *Q* in the Mohr plane $\sigma_{n}-\tau_{n}$. Then an associated flow rule with the nonlinear power-law failure criterion can be established. Further, a constitutive relation in an incremental form can be given on the basis of the plastic potential theory as follows [29]:

$${\dot{\varepsilon }}_{ij}=\dot{\lambda }\frac{\partial Q}{\partial \sigma_{ij}}=\dot{\lambda }\frac{\partial F}{\partial \sigma_{ij}}$$ (3)

where $\dot{\lambda }$ is a plasticity constant; ${\dot{\varepsilon }}_{ij}$ is the plastic strain rate component; $\sigma_{ij}$ is the stress component. In this paper, the nonlinear power-law failure criterion and its associated flow rule are utilized to investigate the failure characteristics of the shallow tunnels in layered soils.

## Upper bound limit analysis of the roof supporting pressure

### Roof collapse mechinsm for shallow tunnels in layered soils

**Fig 2:**
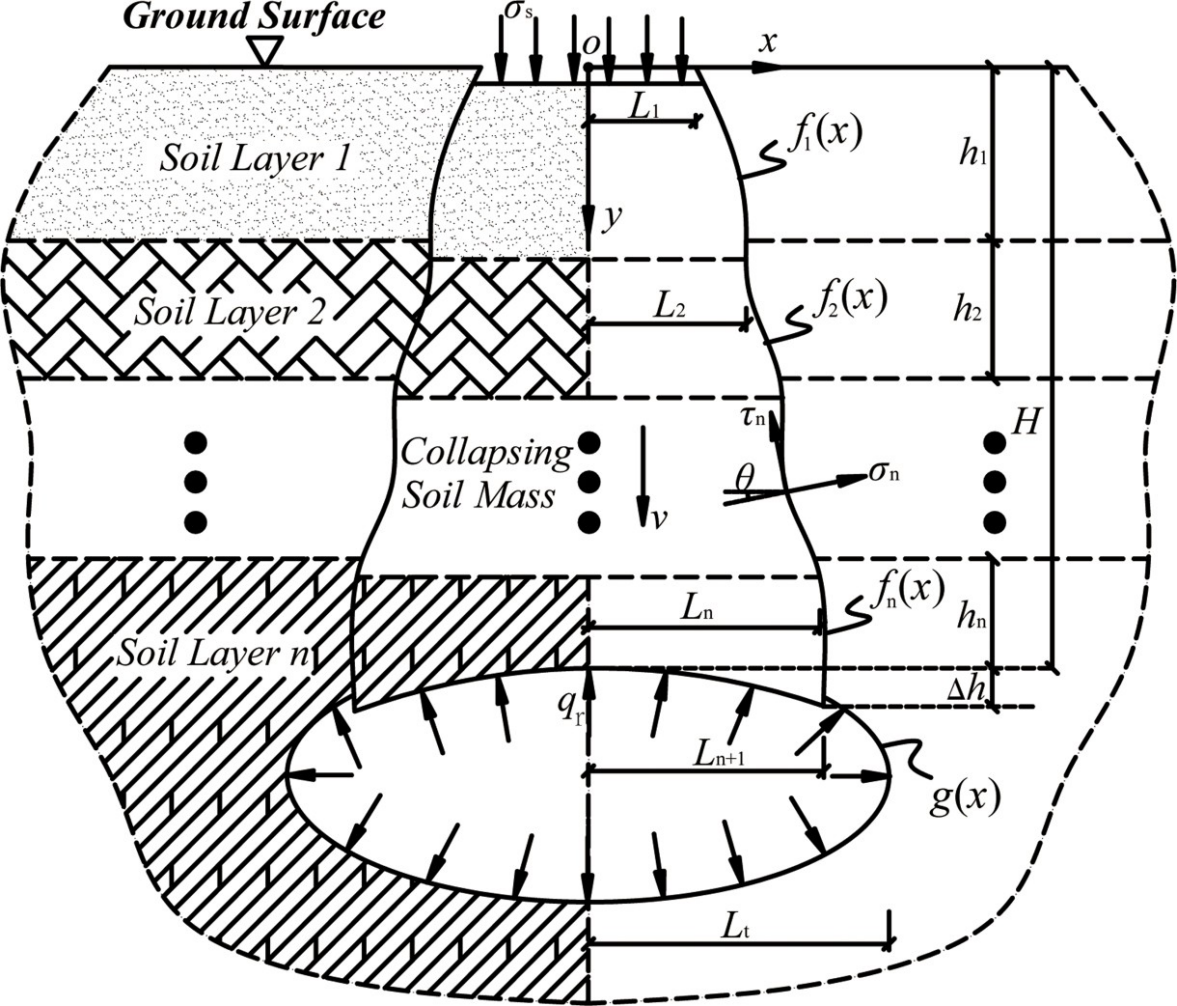
The collapse mechanism of a shallow tunnel with an arbitrary profile in layered soils.

According to the upper bound theorem, a kinematically admissible velocity field, complying with velocity border conditions and deformation compatibility conditions, is required to be built initially for analyzing some failure problems of soils. Only by this way can the virtual work-rate equation be utilized to solve the ultimate load in case of soil failure. As for the shallow tunnels, the soil looseness at the roof will directly reach to the ground surface after tunnel excavation in most cases, due to the smaller thickness of the upper covering soils. Then it is prohibitively difficult to form a collapse arch inside the roof surrounding soil masses, especially under the adverse effect of groundwater. With this failure characteristic into consideration, we propose the corresponding velocity field for a shallow tunnel collapse in layered soils, as shown in Fig 2. The burial depth of the shallow tunnel is *H*. The tunnel profile is arbitrary and its function equation is expressed as g(x). The roof soils are comprised of *n* layers. The collapse curve is comprised of *n* segments, and the expression in soil layer *i* (i = 1, 2, …, *n*) is $f_{i}\left(x\right)$ accordingly. In this velocity field, the roof soil masses are assumed to be an ideal rigid-plastic material. The soil failure obeys the nonlinear power-law failure criterion, as listed in Eq (1). The collapsed soil masses and the surrounding static soil masses are regarded to be rigid, and the downward velocity of the collapsed soil masses is *v*. Thus the internal deformation of the soil masses in these two areas can be ignored. Meanwhile, the effect of the supporting pressure and pore water pressure is incorporated. Particularly, the supporting pressure is assumed to be uniform and perpendicular to the tunnel surface, and its magnitude is $q_{r}$. The pore water pressure in the roof soils can be regarded as an external force that only acts on the failure surfaces of the soil masses. The magnitude of the work rate done by the pore water pressure can be calculated by referring to the proposed method of Viratjandr and Michalowski [30].

### Energy dissipation rate at the soil failure surfaces

Due to the assumption that the collapsed soil masses and the surrounding static soil masses are rigid, the energy dissipation only occurs at the soil failure surfaces. Further we assume the soil failure surface in roof soil layer *i* to be a thin deformable layer with thickness of *w*_*i*_. Accordingly, the plastic strain rates within the thin layer can be obtained on the basis of Eq (3) as follows:

$$\{\begin{array}{l} {\dot{\varepsilon }}_{ni}=-{\dot{\lambda }}_{i}{\left(m_{i}\sigma_{ti}\right)}^{-1}c_{0i}{\left(1+\sigma_{ni}/\sigma_{ti}\right)}^{\left(1-m_{i}\right)/m_{i}}\\ {\dot{\gamma }}_{ni}={\dot{\lambda }}_{i} \end{array}$$ (4)

where ${\dot{\varepsilon }}_{ni}$, ${\dot{\gamma }}_{ni}$ denote the normal and shear strain rate at the failure surface. ${\dot{\lambda }}_{i}$ denotes the plastic constant for layer *i*. $c_{0i}$, $\sigma_{ti}$, $m_{i}$ denote the soil strength parameters for layer *i*.

Meanwhile, according to the geometrical relationship in Fig 2, the plastic strain rates in soil layer *i* can also be expressed as:

$$\{\begin{array}{l} {\dot{\varepsilon }}_{ni}=\left(v/w_{i}\right){\left[1+{f^{\prime }}_{i}{\left(x\right)}^{2}\right]}^{-1/2}\\ {\dot{\gamma }}_{ni}=-\left(v/w_{i}\right){f^{\prime }}_{i}\left(x\right){\left[1+{f^{\prime }}_{i}{\left(x\right)}^{2}\right]}^{-1/2} \end{array}$$ (5)

By combining Eqs (4) and (5), the plastic constant ${\dot{\lambda }}_{i}$ results:

$${\dot{\lambda }}_{i}=-\left(v/w_{i}\right){f^{\prime }}_{i}\left(x\right){\left[1+{f^{\prime }}_{i}{\left(x\right)}^{2}\right]}^{-1/2}$$ (6)

The normal stress at the soil failure surface in layer *i* is:

$$\sigma_{ni}=-\sigma_{ti}+\sigma_{ti}{\left[{\left(m_{i}\sigma_{ti}\right)}^{-1}c_{0i}{f^{\prime }}_{i}\left(x\right)\right]}^{m_{i}/\left(m_{i}-1\right)}$$ (7)

Thus, the magnitude of the internal energy dissipation rate per unit volume in soil layer *i* can be obtained as follows:

$${\dot{D}}_{i}=\sigma_{ni}{\dot{\varepsilon }}_{ni}+\tau_{ni}{\dot{\gamma }}_{ni}=\left\{\left(m_{i}-1\right){\left[m_{i}^{-1}c_{0i}{f^{\prime }}_{i}\left(x\right)\right]}^{m_{i}/\left(m_{i}-1\right)}\sigma_{ti}^{1/\left(1-m_{i}\right)}+\sigma_{ti}\right\}\frac{v}{w_{i}}{\left[1+{f^{\prime }}_{i}{\left(x\right)}^{2}\right]}^{-1/2}$$ (8)

By integrating Eq (8) along the roof collapse curves in *n* soil layers, the total rate of the internal energy dissipation results:

$$W_{D}=\sum_{i=1}^{n}\int_{L_{i}}^{L_{i+1}}\left\{\left(m_{i}-1\right){\left[m_{i}^{-1}c_{0i}{f^{\prime }}_{i}\left(x\right)\right]}^{m_{i}/\left(m_{i}-1\right)}\sigma_{ti}^{1/\left(1-m_{i}\right)}+\sigma_{ti}\right\}dx\cdot v$$ (9)

where the half of the roof collapse block is taken into consideration owing to its symmetry with respect to the *y*-axial. $L_{i}$ is the half width of the collapse block in soil layer *i*.

### Work rates done by external forces

The external forces acting on the collapse block consist of four components: the gravity of soil masses, the supporting pressure, the ground additional load, and the pore water pressure along the detaching curves. The work rate produced by the gravity of soil masses is:

$$W_{\gamma }=\left\{\sum_{i=1}^{n}\gamma_{i}h_{i}L_{i}+\sum_{i=1}^{n}\int_{L_{i}}^{L_{i+1}}\gamma_{i}\left[\sum_{j=1}^{i}h_{j}-f_{i}\left(x\right)\right]dx-\gamma_{n}HL_{n+1}+\int_{0}^{L_{n+1}}\gamma_{n}g\left(x\right)dx\right\}v$$ (10)

where $\gamma_{i}$ is the unit weight for soil layer *i*.

The work rate produced by the supporting pressure is:

$$W_{q}=-\int_{0}^{s}q_{r}cos\alpha ds\cdot v=-\int_{0}^{L_{n+1}}q_{r}\sqrt{1+g^{\prime }{\left(x\right)}^{2}}\cdot \frac{1}{\sqrt{1+g^{\prime }{\left(x\right)}^{2}}}dx\cdot v=-q_{r}L_{n+1}v$$ (11)

where α is the angle between the direction of supporting pressure and the vertical direction at any point of the profile curve *g*(*x*).

The work rate produced by the ground additional load is:

$$W_{\sigma_{s}}=\sigma_{s}L_{1}v$$ (12)

According to Viratjandr and Michalowski [30], the work rate of the pore water pressure done on the tunnel roof is comprised of the following two parts, one on soil particles inside the velocity field and the other on the border of the field. The corresponding expression is listed in the following equation. But, due to the assumption that the collapse block is rigid, the strain rate ${\dot{\varepsilon }}_{ij}$ within the collapse range is equal to zero, which means only the work rate at the detaching surfaces contributes. The corresponding expression can be further simplified as:

$$W_{u}=-\int_{V}u{\dot{\varepsilon }}_{ij}dV-\int_{S}un_{i}vdS=-\sum_{i=1}^{n}\int_{L_{i}}^{L_{i+1}}\gamma_{ui}\gamma_{i}f_{i}\left(x\right)dx\cdot v$$ (13)

where *u* is the pore water pressure at soil failure surface. The $\gamma_{ui}$ is the pore water pressure coefficient in soil layer *i*, The magnitude of the pore water pressure is equal to $\gamma_{ui}\gamma_{i}z$. Specifically, *z* refers to the vertical distance from ground surface to any point of the soil failure surfaces.

### Determination of the roof supporting pressure

Based on the energy dissipation rate and the work rates produced by external forces, the following equation can be given by using virtual work-rate equation.

$$W_{D}=W_{\gamma }+W_{q}+W_{\sigma_{s}}+W_{u}$$ (14)

Substituting Eqs (9)–(13) into Eq (14) results:

$$\begin{array}{l} \sum_{i=1}^{n}\int_{L_{i}}^{L_{i+1}}\left\{\left(m_{i}-1\right){\left[m_{i}^{-1}c_{0i}{f^{\prime }}_{i}\left(x\right)\right]}^{m_{i}/\left(m_{i}-1\right)}\sigma_{ti}^{1/\left(1-m_{i}\right)}+\sigma_{ti}\right\}dx=\sum_{i=1}^{n}\gamma_{i}h_{i}L_{i}+\\ \sum_{i=1}^{n}\int_{L_{i}}^{L_{i+1}}\gamma_{i}\left[\sum_{j=1}^{i}h_{j}-f_{i}\left(x\right)\right]dx-\gamma_{n}HL_{n+1}+\int_{0}^{L_{n+1}}\gamma_{n}g\left(x\right)dx-q_{r}L_{n+1}+\\ \sigma_{s}L_{1}-\sum_{i=1}^{n}\int_{L_{i}}^{L_{i+1}}\gamma_{ui}\gamma_{i}f_{i}\left(x\right)dx \end{array}$$ (15)

According to Eq (15), the required supporting pressure for the shallow tunnels can be expressed as:

$$\begin{array}{c} q_{r}=L_{n+1}^{-1}\sum_{i=1}^{n}\int_{L_{i}}^{L_{i+1}}\Lambda_{i}\left[x,f_{i}\left(x\right),{f^{\prime }}_{i}\left(x\right)\right]dx+L_{n+1}^{-1}\sum_{i=1}^{n}\gamma_{i}h_{i}L_{i}-\gamma_{n}H+\\ L_{n+1}^{-1}\int_{0}^{L_{n+1}}\gamma_{n}g\left(x\right)dx+\sigma_{s}L_{1}L_{n+1}^{-1} \end{array}$$ (16)

where $\Lambda_{i}\left[x,f_{i}\left(x\right),{f^{\prime }}_{i}\left(x\right)\right]$ is:

$$\begin{array}{c} \Lambda_{i}\left[x,f_{i}\left(x\right),{f^{\prime }}_{i}\left(x\right)\right]=\left(1-m_{i}\right){\left[m_{i}^{-1}c_{0i}{f^{\prime }}_{i}\left(x\right)\right]}^{m_{i}/\left(m_{i}-1\right)}\sigma_{ti}^{1/\left(1-m_{i}\right)}-\sigma_{ti}+\\ \gamma_{i}\sum_{j=1}^{i}h_{j}-\left(1+\gamma_{ui}\right)\gamma_{i}f_{i}\left(x\right) \end{array}$$ (17)

It is worth nothing that the collapse of the roof soil masses does not occur instantaneously after tunnel excavation. Instead, the collapse process may exhibit some characteristics of progressive failure. Namely, the magnitude of the collapse scope tends to increase gradually, and the required supporting pressure increases accordingly. Therefore, from the perspective of safe designing in actual engineering, the determination of the required supporting pressure should be based on the most unfavourable failure scenario of the tunnel roof. Specifically, we make the bottom width of the collapse block equating to the tunnel width. Then the collapse range is at its maximum, and it is safest for tunnel support design. The corresponding supporting pressure can be obtained as follows to ensure the long-term stability for tunnel roof.

$$\begin{array}{c} q_{r}=L_{t}^{-1}\sum_{i=1}^{n}\int_{L_{i}}^{L_{i+1}}\Lambda_{i}\left[x,f_{i}\left(x\right),{f^{\prime }}_{i}\left(x\right)\right]dx+L_{t}^{-1}\sum_{i=1}^{n}\gamma_{i}h_{i}L_{i}-\gamma_{n}H+\\ L_{t}^{-1}\int_{0}^{L_{t}}\gamma_{n}g\left(x\right)dx+\sigma_{s}L_{1}L_{t}^{-1} \end{array}$$ (18)

where $L_{t}$ is the half width of the shallow tunnel, and the $L_{i+1}$ (*i* = *n*) is equal to the $L_{t}$.

Eq (18) is a functional of *x* and $f_{i}\left(x\right)$. To obtain the optimal upper bound solution, the following Euler–Lagrange equation is met.

$$\frac{\partial \Lambda_{i}}{\partial f_{i}(x)}-\frac{\partial }{\partial x}\left(\frac{\partial \Lambda_{i}}{\partial {f^{\prime }}_{i}(x)}\right)=0$$ (19)

Substituting Eq (17) into Eq (19) yields a differential equation as follows.

$$-\left(1+\gamma_{ui}\right)\gamma_{i}+\frac{m_{i}}{m_{i}-1}{\left(m_{i}^{-1}c_{0i}\right)}^{m_{i}/\left(m_{i}-1\right)}\sigma_{ti}^{1/\left(1-m_{i}\right)}{\left[{f^{\prime }}_{i}\left(x\right)\right]}^{\left(2-m_{i}\right)/\left(m_{i}-1\right)}{f^{\prime \prime }}_{i}\left(x\right)=0$$ (20)

One integration of Eq (20) yields the $f_{i}{}^{\prime }\left(x\right)$:

$${f^{\prime }}_{i}\left(x\right)=m_{i}\delta_{i}{\left[\frac{A_{i}}{\left(1+\gamma_{ui}\right)\gamma_{i}}+x\right]}^{m_{i}-1}$$ (21)

where $A_{i}$ (i = 1, 2, …, *n*) is an integration constant. The $\delta_{i}$ is:

$$\delta_{i}=c_{0i}^{-m_{i}}\sigma_{ti}{\left[\left(1+\gamma_{ui}\right)\gamma_{i}\right]}^{m_{i}-1}$$ (22)

Further integration of Eq (21) yields the equation $f_{i}\left(x\right)$ of the collapse curve:

$$f_{i}\left(x\right)=\delta_{i}{\left[\frac{A_{i}}{\left(1+\gamma_{ui}\right)\gamma_{i}}+x\right]}^{m_{i}}+B_{i}$$ (23)

where $B_{i}$ (i = 1, 2, …, *n*) is another integration constant.

By substituting Eqs (21) and (23) into Eq (18), the required supporting pressure can be further expressed as:

$$\begin{array}{c} q_{r}=L_{t}^{-1}\sum_{i=1}^{n}\langle -\frac{m_{i}}{m_{i}+1}\delta_{i}\left[\left(1+\gamma_{ui}\right)\gamma_{i}\right]\left\{{\left[\frac{A_{i}}{\left(1+\gamma_{ui}\right)\gamma_{i}}+L_{i+1}\right]}^{m_{i}+1}-{\left[\frac{A_{i}}{\left(1+\gamma_{ui}\right)\gamma_{i}}+L_{i}\right]}^{m_{i}+1}\right\}-\\ \left[\sigma_{ti}-\gamma_{i}\sum_{j=1}^{i}h_{j}+\left(1+\gamma_{ui}\right)\gamma_{i}B_{i}\right]\left(L_{i+1}-L_{i}\right)+\gamma_{i}h_{i}L_{i}\rangle -\gamma_{n}H+L_{t}^{-1}\int_{0}^{L_{t}}\gamma_{n}g\left(x\right)dx+\sigma_{s}L_{1}L_{t}^{-1} \end{array}$$ (24)

Several parameters are unknown in Eq (24). To solve this problem, the results by Yang and Huang [16] can be applied for reference. Specifically, due to the mechanical condition that there is no shear stress at the ground surface, the following equation is met.

$${\tau_{xy}|}_{x=L_{1},y=0}=0$$ (25)

where $\tau_{xy}$ is the shear stress at ground surface. According to the stress equilibrium equation of the element at the junction of the collapse surface and ground surface, the expression of $\tau_{xy}$ can be obtained as follows:

$$\tau_{xy}=\frac{1}{2}\sigma_{n}\sin2\theta -\tau_{n}\cos2\theta =0$$ (26)

where sin2θ, cos2θ can be obtained on the basis of the geometrical relationship that $\cot\theta ={f^{\prime }}_{1}(x)$ in Fig 2 and trigonometric function transformation. Then, by substituting $\sigma_{n}$, $\tau_{n}$ into Eq (26), the constant $A_{1}$ can be determined as:

$$A_{1}=-\left(1+\gamma_{u1}\right)\gamma_{1}L_{1}$$ (27)

Meanwhile, based on the geometric relationships in Fig 2, the following conditions are also met:

$$\left\{ \begin{array}{l} f_{1}\left(x\right){|}_{x=L_{1}}=0\\ f_{2}\left(x\right){|}_{x=L_{2}}=h_{1}\\ f_{3}\left(x\right){|}_{x=L_{3}}=h_{1}+h_{2}\\ \ldots \\ f_{n}\left(x\right){|}_{x=L_{n}}=h_{1}+h_{2}+\ldots +h_{n-1}\\ f_{n}\left(x\right){|}_{x=L_{t}}=g\left(x\right){|}_{x=L_{t}} \end{array}\right.$$ (28)

Then the constant $B_{1}$ is equal to zero. Furtherly, we assume the combined collapse curve are smooth and continuous at the connection points of soil layers, the following equations can be built.

$$\left\{ \begin{array}{l} f_{1}{}^{\prime }\left(x\right){|}_{x=L_{2}}=f_{2}{}^{\prime }\left(x\right){|}_{x=L_{2}}\\ {f^{\prime }}_{2}\left(x\right){|}_{x=L_{3}}={f^{\prime }}_{3}\left(x\right){|}_{x=L_{3}}\\ \ldots \\ {f^{\prime }}_{n-1}\left(x\right){|}_{x=L_{n}}={f^{\prime }}_{n}\left(x\right){|}_{x=L_{n}} \end{array}\right.$$ (29)

$$\left\{ \begin{array}{l} f_{1}\left(x\right){|}_{x=L_{2}}=f_{2}\left(x\right){|}_{x=L_{2}}\\ f_{2}\left(x\right){|}_{x=L_{3}}=f_{3}\left(x\right){|}_{x=L_{3}}\\ \ldots \\ f_{n-1}\left(x\right){|}_{x=L_{n}}=f_{n}\left(x\right){|}_{x=L_{n}} \end{array}\right.$$ (30)

Substituting Eqs (21) and (23) into Eqs (29) and (30) yields the expressions of the constants $A_{i+1}$, $B_{i+1}$(i = 1,2,3…n-1):

$$\left\{ \begin{array}{l} A_{i+1}={\left(m_{i}m_{i+1}^{-1}\delta_{i}\delta_{i+1}^{-1}\right)}^{1/\left(m_{i+1}-1\right)}\left(1+\gamma_{ui+1}\right)\gamma_{i+1}{\left[\frac{A_{i}}{\left(1+\gamma_{ui}\right)\gamma_{i}}+L_{i+1}\right]}^{\left(m_{i}-1\right)/\left(m_{i+1}-1\right)}-\left(1+\gamma_{ui+1}\right)\gamma_{i+1}L_{i+1}\\ B_{i+1}=\delta_{i}{\left[\frac{A_{i}}{\left(1+\gamma_{ui}\right)\gamma_{i}}+L_{i+1}\right]}^{m_{i}}+B_{i}-\delta_{i+1}{\left[\frac{A_{i+1}}{\left(1+\gamma_{ui+1}\right)\gamma_{i+1}}+L_{i+1}\right]}^{m_{i+1}} \end{array}\right.$$ (31)

It should be noted that the Eq (31) denotes the relationships between $A_{i+1}$, $B_{i+1}$ and $A_{i}$, $B_{i}$. The number range of *i* in $A_{i+1}$, $B_{i+1}$ in Eq (31) is 1 to *n*-1, namely the $A_{2}$, $A_{3}$ … $A_{n}$, and $B_{2}$, $B_{3}$ … $B_{n}$. The constants $A_{1}$ and $B_{1}$ have been obtained in Eqs (27) and (28). Thus, by substituting $A_{1}$, $B_{1}$ into Eq (31), the $A_{2}$, $B_{2}$ can be obtained. And the constants $A_{3}$, $A_{4}$ … $A_{n}$, and $B_{3}$, $B_{4}$ … $B_{n}$ can also be obtained by this method in turn. Besides, combining Eqs (28)–(31) can also yield the values of the half widths $L_{1}$, $L_{2}$ … $L_{n}$ of the collapse block in each soil layer. Accordingly, the roof collapse scope corresponding to the required supporting pressure can be determined. The weight of the collapse block can also be obtained as follows:

$$\begin{array}{c} G_{\gamma }=\sum_{i=1}^{n}\gamma_{i}h_{i}L_{i}+\sum_{i=1}^{n}\int_{L_{i}}^{L_{i+1}}\gamma_{i}\left[\sum_{j=1}^{i}h_{j}-f_{i}\left(x\right)\right]dx-\gamma_{n}HL_{t}+\int_{0}^{L_{t}}\gamma_{n}g\left(x\right)dx\\ =\sum_{i=1}^{n}\langle \gamma_{i}\left(\sum_{j=1}^{i}h_{j}-B_{i}\right)\left(L_{i+1}-L_{i}\right)-{\left(m_{i}+1\right)}^{-1}\gamma_{i}\delta_{i}\\ \left\{{\left[\frac{A_{i}}{\left(1+\gamma_{ui}\right)\gamma_{i}}+L_{i+1}\right]}^{m_{i}+1}-{\left[\frac{A_{i}}{\left(1+\gamma_{ui}\right)\gamma_{i}}+L_{i}\right]}^{m_{i}+1}\right\}+\gamma_{i}h_{i}L_{i}\rangle \\ -\gamma_{n}HL_{t}+\int_{0}^{L_{t}}\gamma_{n}g\left(x\right)dx \end{array}$$ (32)

where the width $L_{i+1}$ (*i* = *n*) is equal to the half tunnel width $L_{t}$.

## Discussions and analysis

### Influence of varying parameters on roof supporting pressure

Based on the deduced equations in the preceding section, the required supporting pressure to maintain the roof stability in shallow tunnels can be obtained. This solution is closer to the actual tunnel construction conditions by incorporating the effect of pore water pressure, stratified characteristics of the overlying soils, and arbitrary roof profiles. To further investigate the influence of such factors on the magnitude of the required supporting pressure, a shallow tunnel in two soil layers is selected as an example in the following section. It should be noted that the profile of this tunnel could be circular, elliptical, and rectangular based on the mechanism in Fig 2. For a rectangular tunnel, the expression of the roof profile is:

g(x)=H (33)

By substuting Eq (33) into Eq (24) and setting *n* = 2, the required supporting pressure for a rectangular tunnel in two soil layers can be obtained as follows:

$$\begin{array}{c} q_{r}=L_{t}^{-1}\sum_{i=1}^{2}\langle -\frac{m_{i}}{m_{i}+1}\delta_{i}\left[\left(1+\gamma_{ui}\right)\gamma_{i}\right]\left\{{\left[\frac{A_{i}}{\left(1+\gamma_{ui}\right)\gamma_{i}}+L_{i+1}\right]}^{m_{i}+1}-{\left[\frac{A_{i}}{\left(1+\gamma_{ui}\right)\gamma_{i}}+L_{i}\right]}^{m_{i}+1}\right\}-\\ \left[\sigma_{ti}-\gamma_{i}\sum_{j=1}^{i}h_{j}+\left(1+\gamma_{ui}\right)\gamma_{i}B_{i}\right]\left(L_{i+1}-L_{i}\right)+\gamma_{i}h_{i}L_{i}\rangle +\sigma_{s}L_{1}L_{t}^{-1} \end{array}$$ (34)

where the unknown constants can be calculated by using Eqs (25)–(31).

**Fig 3:**
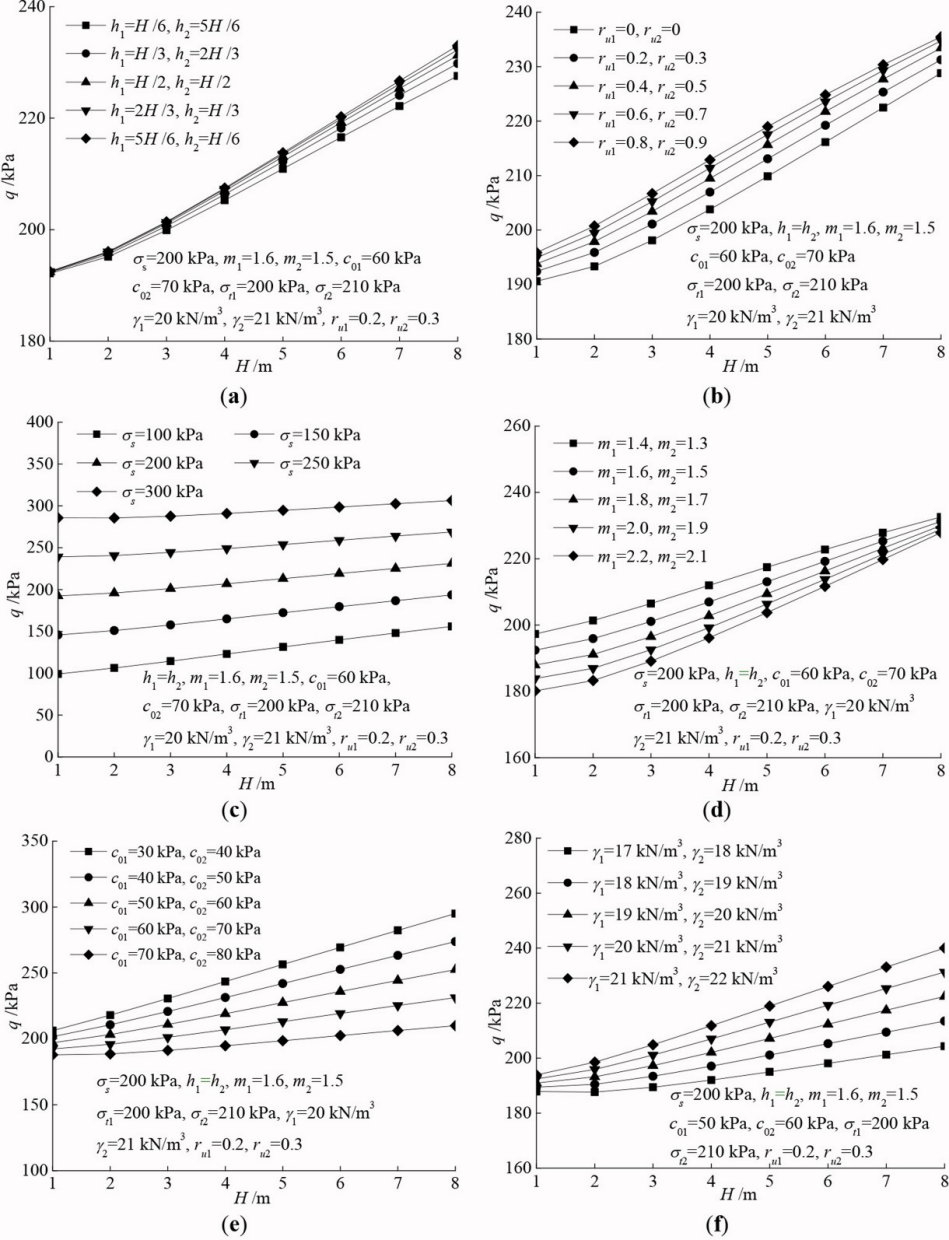
Comparison of the required supporting pressure versus varying parameters. (a) thickness of varying soil layers; (b) pore water pressure coefficient; (c) ground additional load; (d) nonlinear coefficient; (e) soil cohesion; (f) soil unit weight.

Furtherly, the following parameters are chosen for analysis: $h_{1}=H/6-5H/6$, $h_{2}=5H/6-H/6$, $r_{u1}=0-0.8$, $r_{u2}=0-0.9$, $m_{1}=1.4-2.2$, $m_{2}=1.3-2.1$, $c_{01}=30-70\text{kPa}$, $c_{02}=40-80\text{kPa}$, $\gamma_{1}=17-21{\text{kN/m}}^{\text{3}}$, $\gamma_{2}=18-22{\text{kN/m}}^{\text{3}}$, $\sigma_{s}=100-250\text{kPa}$. Fig 3 shows the change law of the required supporting pressure with respect to varying tunnel burial depth and other parameters.

It can be observed from Fig 3 that the required supporting pressure increases with the increase of burial depth. Specifically, due to the strength parameters of the lower soil layer are larger than that of the upper layer in the whole, the supporting pressure decreases as the thickness of the lower layer increases. Especially when the burial depth is bigger, the effect of the thickness of varying soil layers tends to be more obvious. Similarly, the greater values of $c_{0}$ and *m*, the higher soil strength grade, and the smaller required supporting pressure. Nevertheless, when the pore water pressure, ground additional load and soil unit weight are increasing, the magnitude of the supporting pressure tends to increase. These observations are consistent with the results in existing literatures mentioned above.

Furthermore, notice that the effect of the pore water pressure, ground additional load, and soil cohesion on the supporting pressure is more obvious. Particular attention should be paid on such factors in actual tunnel design and construction process. More specific, the following recommendations for the shallow tunnels can be utilized to ensure the safety of engineering construction.

1. The adverse effect of the ground water should be incorporated into the calculation of surrounding soil pressure and the design of roof support, especially when the tunnels pass through groundwater-developed strata. In this way, the support design for shallow tunnels can be more practical for engineering.
2. In order to mitigate the adverse influence of the additional load at ground surface, all kinds of facilities on ground at construction site should be removed in good time. Meanwhile, the damage effect of the ground traffic vibration on tunnel surrounding soils or support structures should be suppressed as far as possible.
3. Increasing the soil strength can effectively reduce the magnitude of the required supporting pressure for shallow tunnels. When tunnels are constructed in soft or weak geology stratum, the techniques such as advanced pipe grouting, bolt grouting, etc. can be employed to reinforce the stratum and enhance the soil strength.

### Influence of varying parameters on roof collapse curves

**Fig 4:**
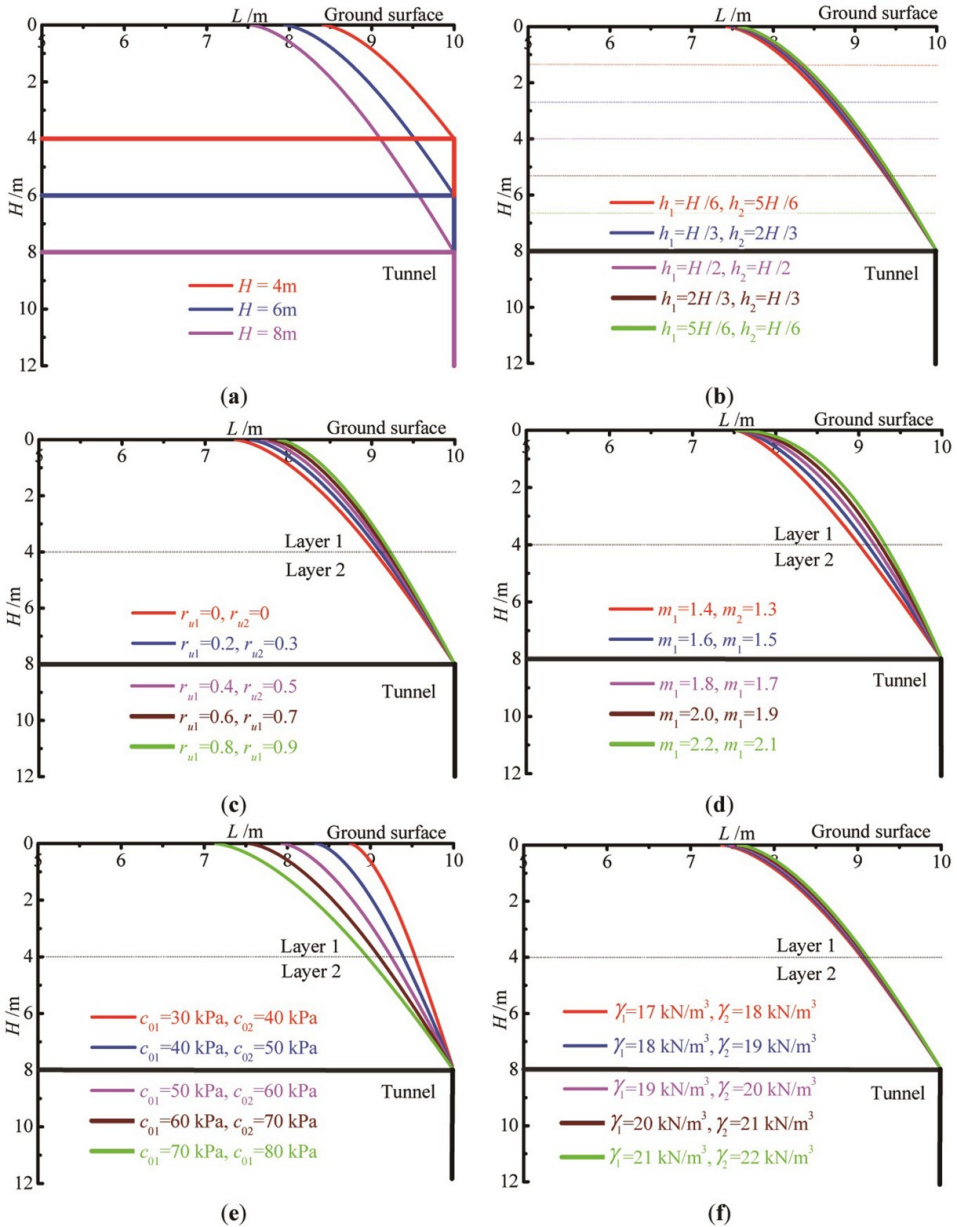
Comparison of the collapse curves versus varying parameters. (a) burial depth; (b) thickness of varying soil layers; (c) pore water pressure coefficient; (d) nonlinear coefficient; (e) soil cohesion; (f) soil unit weight.

According to the preceding analysis, when the optimal upper bound solution of the required supporting pressure is obtained, the equations of the corresponding roof collapse curve can also be determined. To investigate the change law of the collapse curves, the parameters identical to section 4.1 are adopted in this section. The collapse curves in a shallow rectangular tunnel corresponding to varying parameters are plotted, as shown in Fig 4. It can be seen from Fig 4 that the magnitude of roof collapse range is positively related to the burial depth *H* of the shallow tunnel, pore water pressure coefficient *r*_u_, nonlinear coefficient *m*, and soil unit weight *γ*. Conversely, it is negatively related to the thickness of lower soil layer *h*_2_, soil cohesion *c*_0_. Specifically, the effect of the tunnel burial depth, pore water pressure, and soil cohesion is more obvious. The magnitude of the nonlinear coefficient *m* indicates the curvature of collapse curve. The greater value of *m*, the bigger curvature of the collapse curves. When *m* is smaller, the collapse curve tends to be linear.

## Conclusions

1. In this paper, focus is placed on the prediction of the required supporting pressure for the shallow tunnels in layered soils. A curved roof collapse mechanism with multi-failure surfaces is proposed. The analytical solution of the roof supporting pressure is deduced based on upper bound theorem. Specifically, a nonlinear power-law failure criterion is employed to describe the failure characteristics of roof soils.
2. The effect of the number and thickness of soil layers, pore water pressure, roof profile, and ground additional load is incorporated into the expression of the supporting pressure. Thus the proposed method in this paper is closer to the actual tunnel construction conditions by incorporating such factors simultaneously. Furtherly, the corresponding recommendations in actual tunnel design and construction process are also given, which can serve as a guiding theory for support design of shallow tunnels in layered soils.
3. A shallow rectangular tunnel in two soil layers is selected for parametric investigation. The change law of the required supporting pressure and corresponding collapse curves under varying parameters is obtained. We observe that the magnitude of the supporting pressure and roof collapse range are positively related to tunnel burial depth, pore water pressure, and soil unit weight, but negatively related to soil cohesion and thickness of the soil layer with higher strength.
4. The nonlinear coefficient of the soil mass indicates the curvature of the collapse curve. The greater value of it, the bigger curvature and collapse range accordingly, but the smaller supporting pressure is required. Furthermore, the effect of tunnel burial depth, pore water pressure, ground additional load, and soil cohesion is relatively more obvious. These results are consistent with that from the published literatures.

## List of notations

i: number of the soil layer
$\sigma_{n}$, $\sigma_{ni}$: normal stress at the failure surface
$\tau_{n}$, $\tau_{ni}$: shear stress at the failure surface
$c_{0}$, $c_{0i}$: initial cohesion of the soil mass
$\sigma_{t}$, $\sigma_{ti}$: tensile strength of the soil mass
m, $m_{i}$: nonlinear coefficient
F: yield function
Q: plastic potential function
$\dot{\lambda }$, ${\dot{\lambda }}_{i}$: plasticity constant
${\dot{\varepsilon }}_{ij}$: plastic strain rate component
$\sigma_{ij}$: stress component
${\dot{\varepsilon }}_{ni}$: normal strain rate at the failure surface
${\dot{\gamma }}_{ni}$: shear strain rate at the failure surface
H: burial depth of the shallow tunnel
g(x): curve equation of the tunnel profile
$f_{i}\left(x\right)$: curve equation of the collapse curve in soil layer i
v: collapsing velocity
$q_{r}$: supporting pressure
$w_{i}$: thickness of the thin deformable layer in soil layer i
${f^{\prime }}_{i}\left(x\right)$: derivative of $f_{i}\left(x\right)$
${\dot{D}}_{i}$: internal energy dissipation rate per unit volume in soil layer i
$W_{D}$: total rate of the internal energy dissipation
$L_{i}$: half width of the collapse block in soil layer i
$W_{\gamma }$: work rate produced by the gravity of soil masses
$\gamma_{i}$: unit weight for soil layer i
$W_{q}$: work rate produced by the supporting pressure
$\sigma_{s}$: ground additional load
$W_{\sigma_{s}}$: work rate produced by the ground additional load
$W_{u}$: work rate produced by the pore water pressure
u: pore water pressure
u: pore water pressure coefficient
z: vertical distance from ground surface to any point of the failure surfaces
$L_{t}$: half width of the shallow tunnel
$A_{i}$, $B_{i}$: integration constant
$G_{\gamma }$: weight of the collapsing soil masses
